# Moringa (*Moringa oleifera*) green-synthesized copper oxide nanoparticles for the drought tolerance of tomato (*Solanum lycopersicum*)

**DOI:** 10.1186/s12870-025-06708-2

**Published:** 2025-05-23

**Authors:** Rania El-Tanbouly, Mahmoud A. Gaber, Sara Omran, Nada yahia Ahmed, Alaa Nader Ali, Asmaa Hassan Saleh, Aya Mohamed Ramadan Elgamal, Nadin Khafaji, Sarah EL-Messeiry

**Affiliations:** 1 Department of Floriculture, Ornamental Horticulture and Landscape Design, Faculty of Agriculture, Alexandria University, Alexandria, 21545 Egypt; 2 Department of Plant Pathology, Faculty of Agriculture (El-Shatby), Alexandria University, Alexandria, 21545 Egypt; 3 Department of Genetics, Faculty of Agriculture (El-Shatby), Alexandria University, Alexandria, 21545 Egypt

**Keywords:** Nanoparticle, Moringa, Drought, Tomato, Plant extract, qRT‒PCR, Stress genes

## Abstract

The increasing impacts of climate change, global warming, and water scarcity are intensifying drought risk and compromising global food security. To mitigate these challenges, green-synthesized nanoparticles (NPs) derived from plant extracts have recently emerged as an innovative tool for enhancing crop resilience to abiotic stresses such as drought. Moringa enables eco-friendly nanoparticle production with diverse uses. Although Moringa enhances plant traits and its NPs have antimicrobial properties, limited research exists on their ability to improve plant tolerance to abiotic stress. Here, we investigated the potential of copper oxide (CuO) nanoparticles synthesized from Moringa plant extracts to alleviate drought stress in tomato plants (*Solanum lycopersicum*). The Moringa-based CuO NPs were characterized via UV‒Vis spectrophotometry, Zeta potential, FTIR, SEM, and TEM. Foliar applications of the NPs at various concentrations (3, 6, and 9 mg/L) were tested on tomato seedlings under normal and drought-induced conditions (10% PEG), alongside two controls: non-stressed control (water treatment) and drought stress control (PEG treatment). Physiological assessments showed that 6 mg/L Moringa-based CuO NPs were most effective under drought stress, significantly reducing leaf yellowing and increasing shoot length by 11%, root length by 21%, total dry biomass by 34% increase, and total chlorophyll content by 32% increase at (*p* < 0.05) compared to the drought stress control. Furthermore, at the molecular level, gene expression analysis revealed the upregulation of *PAL*, *CHS*, and *HQT*, which are genes critical to the stress response and secondary metabolism in tomato plants. Notably, the combination of CuO NPs with PEG amplified gene expression, indicating a synergistic effect on improving drought tolerance. These findings highlight the potential of the use of Moringa-synthesized CuO nanoparticles as a sustainable, eco-friendly strategy to mitigate drought stress in economic crops such as tomatoes. This new green approach offers a promising solution for bolstering food security in the face of climate change and water scarcity challenges.

**Supplementary Information:**

The online version contains supplementary material available at 10.1186/s12870-025-06708-2.

## Introduction

The global population is projected to reach 9.6 billion by 2050, necessitating a substantial increase in agricultural production to meet rising food demands. this urges the adoption of innovative and sustainable agricultural strategies to ensure global sustainability food security [1, 2]. Tomato (*Solanum lycopersicum*), a member of the Solanaceae family, is among the most widely cultivated and economically important crops worldwide. The rich nutritional profile—comprising ascorbic acid, flavonoids, lycopene, and β-carotene—tomatoes are integral to human diets and global agriculture [3]. However, their cultivation faces increasing threats from adverse environmental conditions, including drought, salinity, flooding, and temperature extremes. These challenges are further exacerbated by the growing impacts of climate change [4].

Drought, in particular, poses a critical challenge to agricultural productivity because of its profound effects on crop growth and development. Approximately 40% of the global agricultural output depends on irrigated land, highlighting the significant relationship between water availability and food production [5]. Water, comprising 80–95% of a plant’s fresh biomass, plays an essential role in physiological processes crucial for plant growth, such as nutrient uptake, photosynthesis, and cellular expansion [6, 7]. Drought stress disrupts these critical physiological processes, resulting in significant reductions in both crop yield and overall biomass production. With the increasing frequency and intensity of drought events due to anthropogenic climate change, strategies to increase crop resilience to water scarcity are urgently needed [8].

Tomato plants are highly susceptible to drought stress, which significantly impacts their growth, yield, and overall health worldwide. Drought stress disrupts essential physiological processes such as photosynthesis, nutrient uptake, and water regulation, leading to reduced fruit quality and quantity. According to studies, drought conditions trigger oxidative stress in tomato plants, causing cellular damage and impairing metabolic functions [9–11]. Additionally, water scarcity exacerbates the vulnerability of tomato crops to pests and diseases, further compromising productivity. Efforts to develop drought-resistant tomato and implement sustainable irrigation practices are crucial to mitigate these challenges and ensure global food production resilience.

Nanotechnology has emerged as a promising solution to mitigate the impacts of drought and other abiotic stresses on crops. The unique properties of nanomaterials, such as their high surface area-to-volume ratio and enhanced reactivity, enable a range of applications aimed at improving plant resilience and productivity under challenging conditions [4]. Among the various methods of nanoparticle synthesis, plant-mediated or “green synthesis” approaches are particularly appealing because of their cost-effectiveness, biocompatibility, and environmental sustainability. This method utilizes natural plant compounds to synthesize nanoparticles that exhibit superior stability and reduced toxicity compared to those produced via traditional physical and chemical techniques [12, 13].

Copper oxide nanoparticles (CuO NPs) have garnered attention in agricultural research for their potential to increase plant growth and abiotic stress tolerance. Their high surface area and ability to penetrate plant tissues make them effective at facilitating nutrient absorption and improving plant resilience. Studies have shown that CuO NPs promote osmotic adjustment and the accumulation of protective osmolytes under drought and salinity stress while increasing the chlorophyll content and photosynthesis-related enzyme activity [14, 15]. These properties result in improved photosynthetic efficiency, greater biomass accumulation, and higher crop yields, highlighting their potential as tools for addressing the challenges of climate change and water scarcity.

*Moringa oleifera* (Moringa) leaves are rich in polyphenols, which play a crucial role in the green synthesis of nanoparticles. These polyphenols act as powerful reducing agents, facilitating the conversion of metal ions to nanoparticles. This process not only enhances the efficiency of nanoparticle formation but also contributes to the stability of the resulting particles. Moreover, the use of these naturally occurring compounds aligns with environmentally friendly synthesis methods, reducing the need for harsh chemicals [16]. Additionally, Moringa nanoparticles have shown great promise in enhancing agricultural practices by leveraging the nutrient-rich properties of the Moringa plant via nanotechnology. These nanoparticles, derived from Moringa extract nanoparticles, can act as bionanofertilizers, providing plants with essential nutrients and natural protective compounds in a highly bioavailable form. The antioxidants and phytochemicals in Moringa can contribute to improved plant growth, stress tolerance, and resistance to pathogens [16, 17]. However, the effects of Moringa-synthesized nanoparticles on drought tolerance have not been investigated previously.

This research investigates the use of green-synthesized nanoparticles, leveraging nanotechnology, to enhance crop resilience to drought. We hypothesized that foliar application of Moringa-synthesized copper oxide nanoparticles (CuO NPs) would mitigate drought stress in tomato plants by improving plant physiological performance through the modulation of stress-response genes. This study aims to synthesize and validate Moringa nanoparticles and investigate their impact on the physiology and gene expression of drought- tolerant tomato plants. The expected findings will contribute to sustainable approaches for tomato production and mitigating the impact of drought on economically important crops.

## Materials and methods

### Preparation of plant extracts

*Moringa oleifera* Lam. (Moringa) leaves were collected from a tree located in the faculty of Agriculture, Alexandria University Garden, Egypt. Fully expanded, dark green health leaves were collected and washed with deionized water. Leaves were oven-dried at 60 ^ο^C for approximately 6 h until sufficiently dry for grinding. This drying process aimed to minimize moisture content while avoiding significant loss of bioactive plant compounds [18, 19]. Furthermore, Fourier transform infrared (FTIR) analysis was conducted on the dried leaf powder to ensure the abundance of functional groups and no significant loss occurred during the drying process (Supplementary Material). The dried leaves were homogenized using a mortar and pestle. A total of 10 g of dried leaf powder was added to 100 mL of deionized water in a flask and boiled on a hot plate magnetic stirrer for 30 min. After boiling, the mixture was allowed to cool at room temperature and then filtered using Whatman filter paper grade 42. The filtrate was stored at 4 ^ο^C for nanoparticle synthesis.

### Green synthesis of nanoparticles

**Fig. 1:**
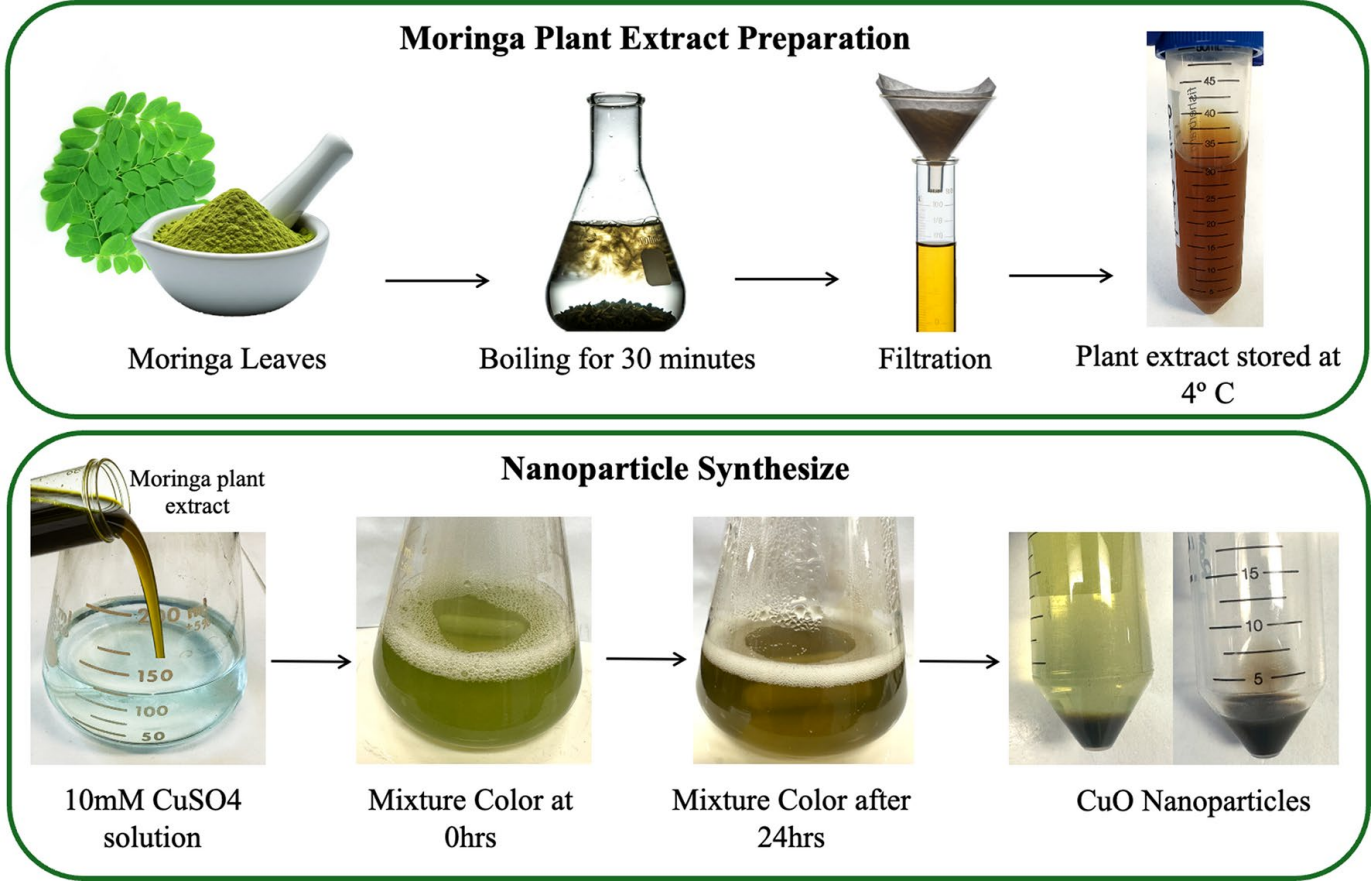
An illustration showing the green synthesis of copper nanoparticles from Moringa plant extract

10 ml of the plant extract was added to 100 ml of 10 mM copper sulfate (CuSO_4_) (Sigma, U.S.A.) and stirred at 700 rpm for 24 h at room temperature until color changes were observed. Following the stirring period, the mixture was centrifuged for 10 min at maximum speed and washed with deionized water to remove excess residue (Fig. 1).

### Nanoparticle characterization

The synthesized nanoparticles were characterized using five different methods to determine particle size and properties: (a) UV-Vis spectrophotometer (Molecular Device SpectraMax M2) and, (b) Fourier transform infrared (FTIR) spectroscopy. FTIR analysis was subsequently conducted on the dried powder form of the synthesized CuO NPs. The sample was dried in the oven at 40 °C for 24 h and then ground into powder before loading on the FTIR spectroscopy machine. (c) Scanning electron microscopy (SEM) (JSM-IT200) employs a high-energy electron beam to scan the sample surface, with electromagnetic condenser and objective lenses to control and focus the beam. (d) Transmission electron microscopy (TEM) (JEM-1400Plus) transmits a high-energy electron beam through an ultrathin specimen. TEM utilizes a series of electromagnetic lenses, including condenser, objective, and projector lenses, to focus and magnify the transmitted electrons, enabling for high-resolution imaging of the internal structure and composition of a sample. (e) Zeta potential analysis was conducted to assess the surface charge and colloidal stability of the nanoparticles in a hydrodynamic environment (Malvern Zetasizer Nano ZS, U.K., at 25 °C). The nanoparticles were suspended in deionized water and subjected to 3 min of sonication, using (DRAWELL Ultra sonicator, U.K.), to ensure uniform dispersion and minimize aggregation. Sonication time was limited to three minutes to avoid the formation of aggregates [20]. All the equipment used was in the electron microscope unit at the Faculty of Science, Alexandria University, Egypt.

### Tomato plant preparation and drought conditions

Thirty-day-old *Solanum lycopersicum* (tomato) variety “Fremont 51012” seedlings were purchased from commercial vendor (Roaia from Agriculture development, Egypt) for the study. These seedlings were carefully transplanted into sterilized soil, which consisted of a 1:1 ratio of peat moss and vermiculite and placed in planting bags. To ensure optimal growing conditions, the plants were subsequently transferred to a greenhouse with meticulous regulation of temperature and humidity, ensuring uniformity throughout. The ambient conditions were maintained at 25 ± 2 °C during the day and 18 ± 2 °C at night, with a relative humidity of 60 ± 5% and a 16-hour photoperiod. After transplantation, the plants were thoroughly irrigated with distilled water until the soil was completely saturated. The tomato plants were acclimated and allowed to adapt for 4 days.

Tomato plants were exposed to 10% (w/v) PEG 6000 (Sigma, U.S.A) for five days to simulate drought conditions by retaining water molecules in the soil [21]. The soil moisture, pH, and temperature were monitored throughout the experiment via a 3-in-1 soil tester (China).

### Nanoparticle treatment

**Fig. 2:**
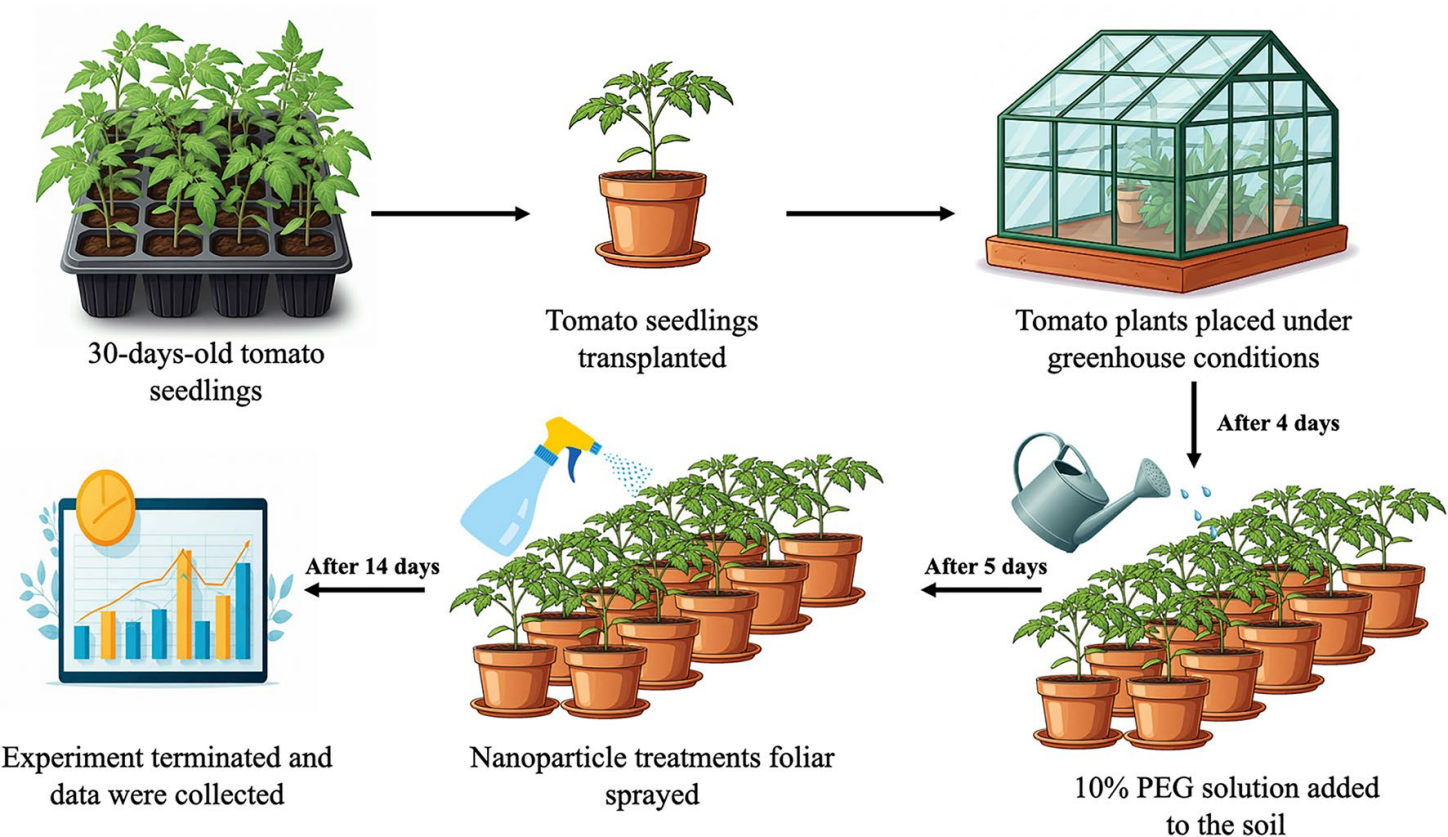
An illustration of the experimental setup. Thirty-day-old tomato seedlings were transplanted, followed by 10% PEG application, nanoparticle treatment, and sampling and measurement

To evaluate the impact of foliar application of green synthesized nanoparticles, the tomato seedlings were divided into eight groups, each with five biological replicates. The treatment groups and conditions are described in Table 1. For each group, the plants were foliar sprayed with the appropriate suspensions as described earlier, ensuring thorough wetting. The CuO NP concentrations used were 0, 3, 6, and 9 mg/L. These low concentrations aimed to evaluate dose-dependent effects while minimizing the risk of phytotoxicity, with the goal of enhancing drought tolerance [22] (Fig. 2).

**Table 1:** Treatments used for the drought experiment

No.	Treatments	Conditions
1	T1	No drought + Water spray
2	T2	No drought + Nanoparticle (3 mg/L)
3	T3	No drought + Nanoparticle (6 mg/L)
4	T4	No drought + Nanoparticle (9 mg/L)
5	D1	Drought (10% PEG) + Water spray
6	D2	Drought (10% PEG) + Nanoparticle (3 mg/L)
7	D3	Drought (10% PEG) + Nanoparticle (6 mg/L)
8	D4	Drought (10% PEG) + Nanoparticle (9 mg/L)

### Plant growth parameters and chlorophyll assay

The experiment was terminated 15 days after the foliar application of the nanoparticles, and comprehensive plant growth data were collected. The parameters measured included the number of green leaves and yellow leaves, as well as an assessment of plant wilting and overall physiological conditions. Shoot and root lengths were recorded, and biomass measurements were taken for both the fresh and dry weights of the shoots and roots. The soil pH was periodically monitored throughout the experimental period to ensure that it remained below 8, maintaining consistent conditions for plant growth. The chlorophyll content was quantified by collecting three representative leaves from each plant for each treatment, which were analyzed via a SPAD-502 Plus chlorophyll meter.

### RNA isolation

For each treatment, 5–9 replicates were used, and the leaves were pooled to create a single biological replicate per treatment with appropriate controls. Approximately 100 mg of fresh tissue was collected and immediately ground to a fine powder using a mortar and pestle with liquid nitrogen to ensure complete cellular disruption. TRIzol reagent (Thermo, U.S.A.) (1 mL) was added to the powdered tissue to prevent RNA degradation, and the mixture was thoroughly homogenized. The homogenate was transferred to an RNase-free microcentrifuge tube and incubated at room temperature for 5 min to allow for the dissociation of nucleoprotein complexes. Following incubation, 200 µL of chloroform (Fisher, U.S.A) was added to the mixture, which was then vortexed vigorously for 15 s and incubated at room temperature for 2–3 min. The samples were centrifuged at 12,000 × g for 15 min at 4 °C, resulting in three distinct phases: an upper aqueous phase containing RNA, an interphase containing DNA, and a lower organic phase containing proteins. The upper aqueous phase was carefully transferred to a new RNase-free tube, avoiding contamination from the interphase. The RNA was precipitated by adding 500 µL of isopropanol to the aqueous phase, followed by gentle mixing and incubation at room temperature for 10 min. The samples were subsequently centrifuged at 12,000 × g for 10 min at 4 °C to pellet the RNA.

The resulting RNA pellet was washed with 1 mL of 75% ethanol (Fisher, U.S.A.), vortexed briefly, and centrifuged at 7,500 × g for 5 min at 4 °C. After the ethanol was removed, the pellet was air-dried for 5–10 min and resuspended in RNase-free water. The RNA concentration and purity were assessed via a NanoDrop spectrophotometer (MAESTRO, Tawiwan), and the samples were stored at − 80 °C for subsequent applications. To assess RNA integrity, 5 µl of each RNA sample was mixed with RNA loading dye (Thermo, U.S.A.), heated to 56 °C, chilled on ice, and analyzed by electrophoresis on a 0.7% (w/v) agarose gel (Thermo, U.S.A). The gel was run at 100 V for 30 min and subsequently stained with 10 µg/mL ethidium bromide for 30 min.

### Gene expression analysis

After RNA verification, 100 ng of each sample was used for quantitative real-time PCR (qRT‒PCR) analysis via the GoTaq 1-Step RT‒qPCR System (Promega, U.S.A.). The reaction mixture was prepared in a final volume of 20 µl according to the manufacturer’s instructions and conducted in triplicate. Table 2 shows the sequences and accession numbers of the primers used [23].

**Table 2:** Primer sequences of phenylpropanoid pathway genes used in this study

Gene	Accession number	Abbreviation	Sequence (5′-3′)
Elongation Factor 1-alpha *(EF1-α)*	AB061263	EF1-α F	ATTGGAAACGGATATGCTCCA
		EF1-α R	TCCTTACCTGAACGCCTGTCA
Phenylalanine Ammonia-Lyase (*PAL*)	BG887005	PAL F	CGGGTTGCCATCTAATCTGACA
		PAL R	CGAGCAATAAGAAGCCATCGCAAT
Chalcone Synthase (*CHS*)	BG888147	CHS F	CACCGTGGAGGAGTATCGTAAGGC
		CHS R	TGATCAACACAGTTGGAAGGCG
Hydroxycinnamoyl-CoA Quinate Hydroxycinnamoyl Transferase (*HQT*)	BF 154,152	HQT F	CCCAATGGCTGGAAGATTAGCTA
		HQT R	CATGAATCACTTTCAGCCTCAACAA

qRT‒PCR was conducted via the STRATAGENE MX3000P real-time PCR system under the following cycling conditions: reverse transcription at 37 °C for 15 min, reverse transcriptase inactivation and GoTaq^®^ DNA polymerase activation at 95 °C for 10 min, followed by 40 cycles of 95 °C for 10 s (denaturation), 60 °C for 30 s (annealing), and 72 °C for 30 s (extension). Relative gene expression was calculated via the 2^−ΔΔCT method via normalization against control samples, and elongation factor 1-alpha *(EF1-α) was used as* a reference gene [24].

### Statistical analysis

Statistical analyses were performed using GraphPad Prism software (version 6.0.1). To determine significant differences between treatment groups, all data were subjected to one-way ANOVA at the LSD 0.05 level. Mean values for experimental groups comparison ranges were compared using Tukey’s test. Differences were considered statistically significant at *p* < 0.05. A randomized complete block design (RCBD) was conducted with 5 biological replicates in each of the 8 groups described in Table 1.

## Results

### Validation and characterization of the synthesized nanoparticles

**Fig. 3:**
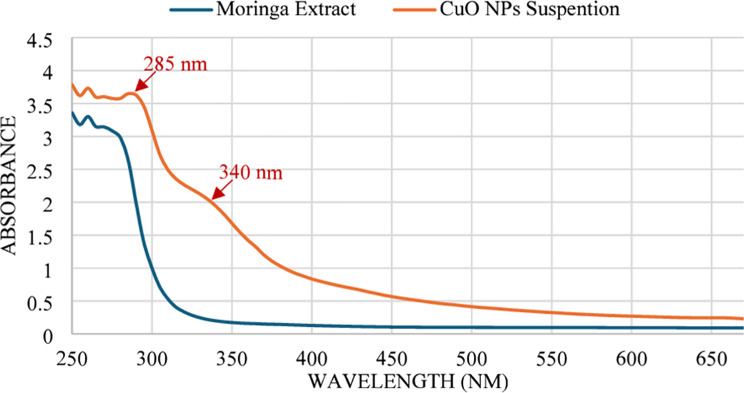
UV‒Vis spectrophotometry of the Moringa plant extract and CuO NPs

The synthesized copper oxide nanoparticles were analyzed via a UV‒Vis spectrophotometer to verify the presence of surface plasmon resonance within the range of 250–700 nm (Fig. 3). The results revealed two peaks at 285 and 340 nm in the CuO NP suspension, which corresponds to the SPR absorption band of the CuO nanoparticles and did not exist in the plant extract solution, confirming their formation. As the wavelength increased, the intensity of the absorbance decreased, suggesting that significant formation did not take place at longer wavelengths. The absorbance feature observed at 340 nm is a broad band rather than a sharp peak, which is characteristic of CuO NPs as reported in previous studies [25–27]. Additional peaks were observed near 250 nm in both the Moringa extract and CuO NPs suspension, suggesting the presence of residual plant extract components associated with the synthesized nanoparticles. This observation aligns with previous research indicating that CuO nanoparticles typically form within the range of 200–350 nm [28–31].

**Fig. 4:**
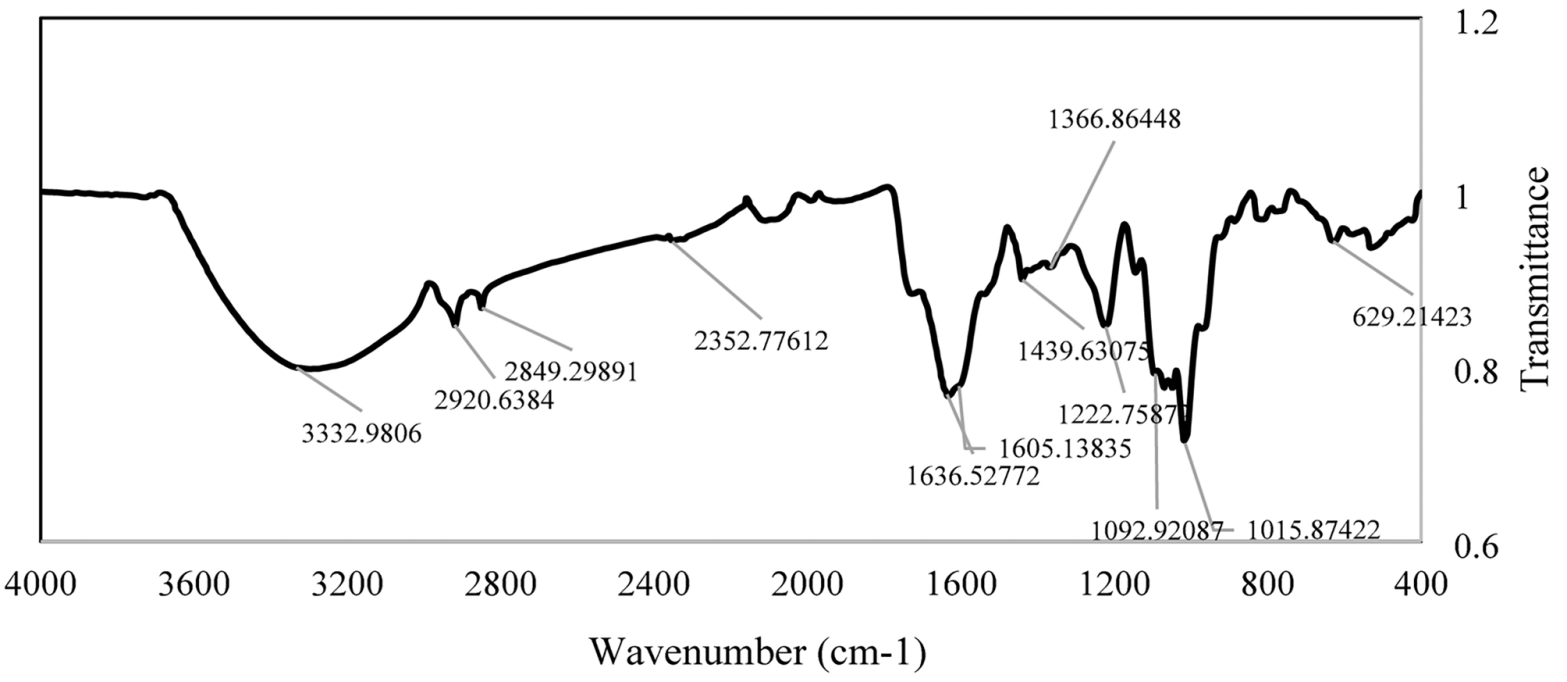
FTIR spectrum of synthesized CuO NPs showing absorption peaks

The functional groups present in the dry CuO NP sample were identified via FTIR spectroscopy in the range of 4000–400 cm^− 1^ (Table 3; Fig. 4). FTIR spectral analysis revealed characteristic absorption peaks corresponding to various functional groups in the CuO NPs. The strong and broad peak at 3332.98 cm⁻¹ is attributed to O-H stretching, which is indicative of hydroxyl groups. The peak at 2920.64 cm⁻¹ corresponds to C–H stretching, which is characteristic of alkyl groups. The distinct absorption at 1636.53 cm⁻¹ is assigned to C = C stretching, indicating the presence of alkenes or aromatic compounds. The peak at 1439.63 cm⁻¹ signifies O-H bending, which is typically associated with alcohols or phenols. The presence of sulfur-containing groups is confirmed by the S = O stretching peak at 1366.86 cm⁻¹. The peaks at 1222.76 cm⁻¹ and 1092.92 cm⁻¹ are attributed to C-O stretching, which is consistent with the presence of esters, ethers, or alcohols. Finally, the peak at 1015.87 cm⁻¹ corresponds to C‒F stretching, indicating fluorine-containing functional groups. These results provide a comprehensive identification of the functional groups present in CuO NPs, reflecting their complex chemical composition.

**Table 3:** Absorption peaks of the prepared Cu/CuO nanoparticles obtained from the FTIR spectrophotometer and their corresponding groups [32]

No.	Absorption Peak Position (Wavenumber cm^− 1^)	Functional Group*
1	3332.98	O-H stretching
2	2920.64	C-H stretching
3	1636.53	C = C stretching
4	1439.63	O-H bending
5	1366.86	S = O stretching
6	1222.76	C-O stretching
7	1092.92	C-O stretching
8	1015.87	C-F stretching

*FTIR Functional Group Database Table with Search InstaNANO

The nanoparticles were detected and analyzed via a scanning electron microscope (SEM) located in the electron microscope unit at the Faculty of Science, Alexandria University, Egypt. This analysis was conducted on samples of Moringa plants loaded with copper sulfate.

**Fig. 5:**
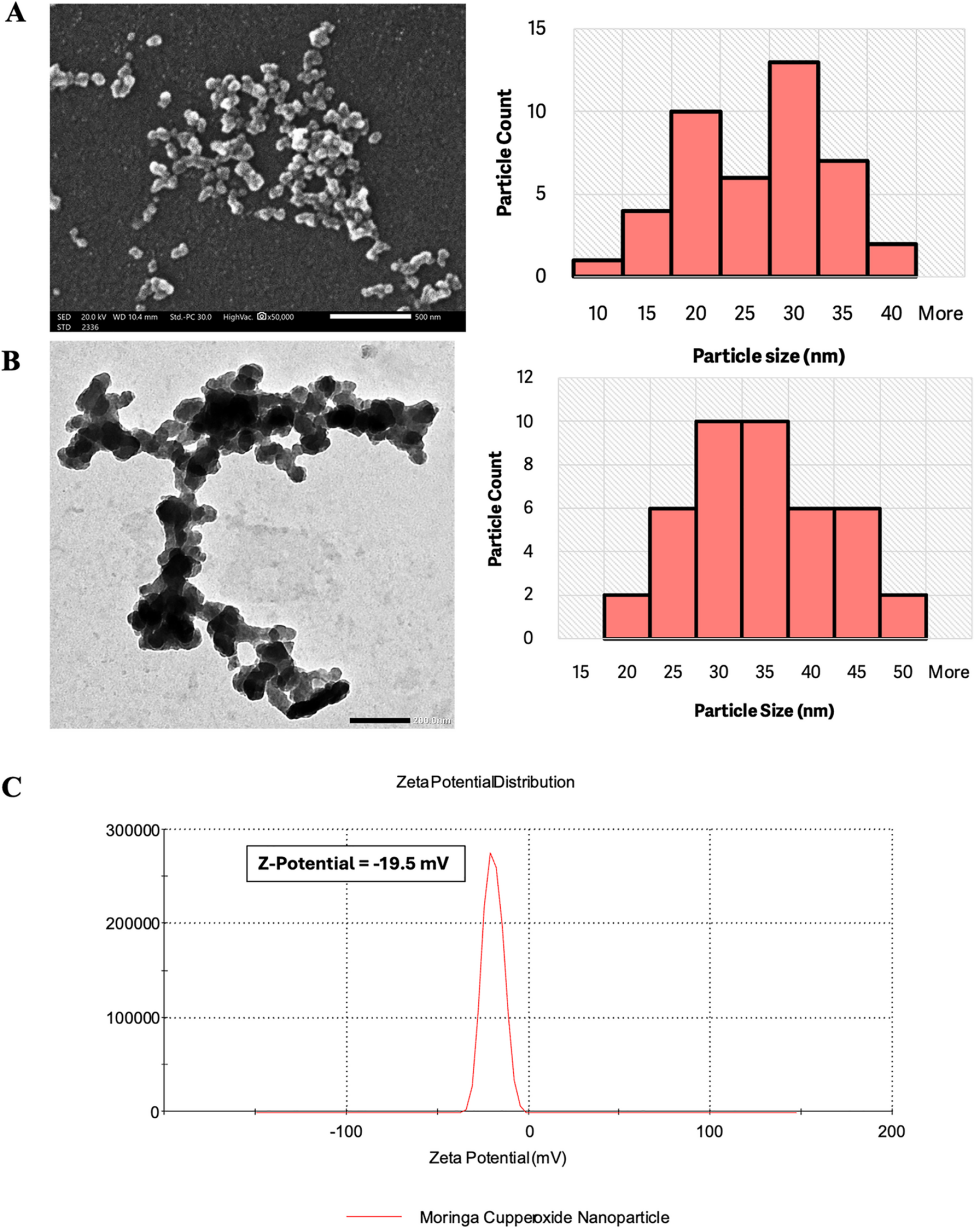
Electron microscopy images and particle size distributions of the nanoparticle aggregates. (**A**) SEM image (35,000×) showing loosely packed aggregates (scale: 500 nm) and the corresponding particle size distribution with slight asymmetry. (**B**) TEM image showing densely packed aggregates (scale: 200 nm) and a symmetrical particle size distribution. (**C**) The zeta potential of green synthesized copper oxide nanoparticles with negative charge (mV) indicating a stable dispersion due to the repulsive electrostatic force between particles

The average particle size of the moringa samples ranged between 26 and 33 nm. These findings are summarized in Fig. 5. A, which presents the average sizes of the nanoparticles detected in moringa plant samples. The use of SEM analysis allowed for the precise characterization of the nanoparticle sizes present in the treated moringa plant materials, providing valuable insights into the properties of the nanomaterials used in this study.

**Table 4:** Average size of the Moringa nanoparticles detected via SEM and TEM

Detection EM	Average Particle Size (nm) (± SD)
SEM	23.9(± 7.387)
TEM	32.07(± 7.997)

The nanoparticles were detected via transmission electron microscopy (TEM) using the electron microscope facility at the Faculty of Science, Alexandria University, Egypt. This analysis was conducted on samples of Moringa plants loaded with copper sulfate.

The average particle size of the moringa samples ranged between 26 and 55 nm. These findings are summarized in Fig. 5B, which presents the average sizes of the nanoparticles detected in the Moringa plant samples. The use of TEM analysis allowed for the precise characterization of the nanoparticle sizes present in the treated Moringa plant materials, providing valuable insights into the properties of the nanomaterials used in this study. The average sizes of the synthesized Moringa-based CuO nanoparticles, as determined by SEM and TEM (Table 4), were 23.9 nm and 32.07 nm, respectively, with no significant differences observed at p-value (0.005).

Furthermore, the zeta potential analysis revealed a negative charge of -19.5 mV, indicating a stable dispersion of the synthesized Moringa CuO nanoparticles due to the repulsive electrostatic forces between the particles, as represented in Fig. 5C.

The comprehensive characterization results, including UV-Vis spectroscopy, FTIR analysis, electron microscopy (SEM and TEM), and zeta potential measurements, collectively demonstrate the successful green synthesis of copper oxide nanoparticles using Moringa aqueous plant extract. The synthesized CuO NPs exhibit consistent size distributions, stable dispersion, and a complex surface chemistry, validating the efficacy of this eco-friendly synthesis method.

### Effects of nanoparticle treatments on tomato plant growth under drought stress

**Fig. 6:**
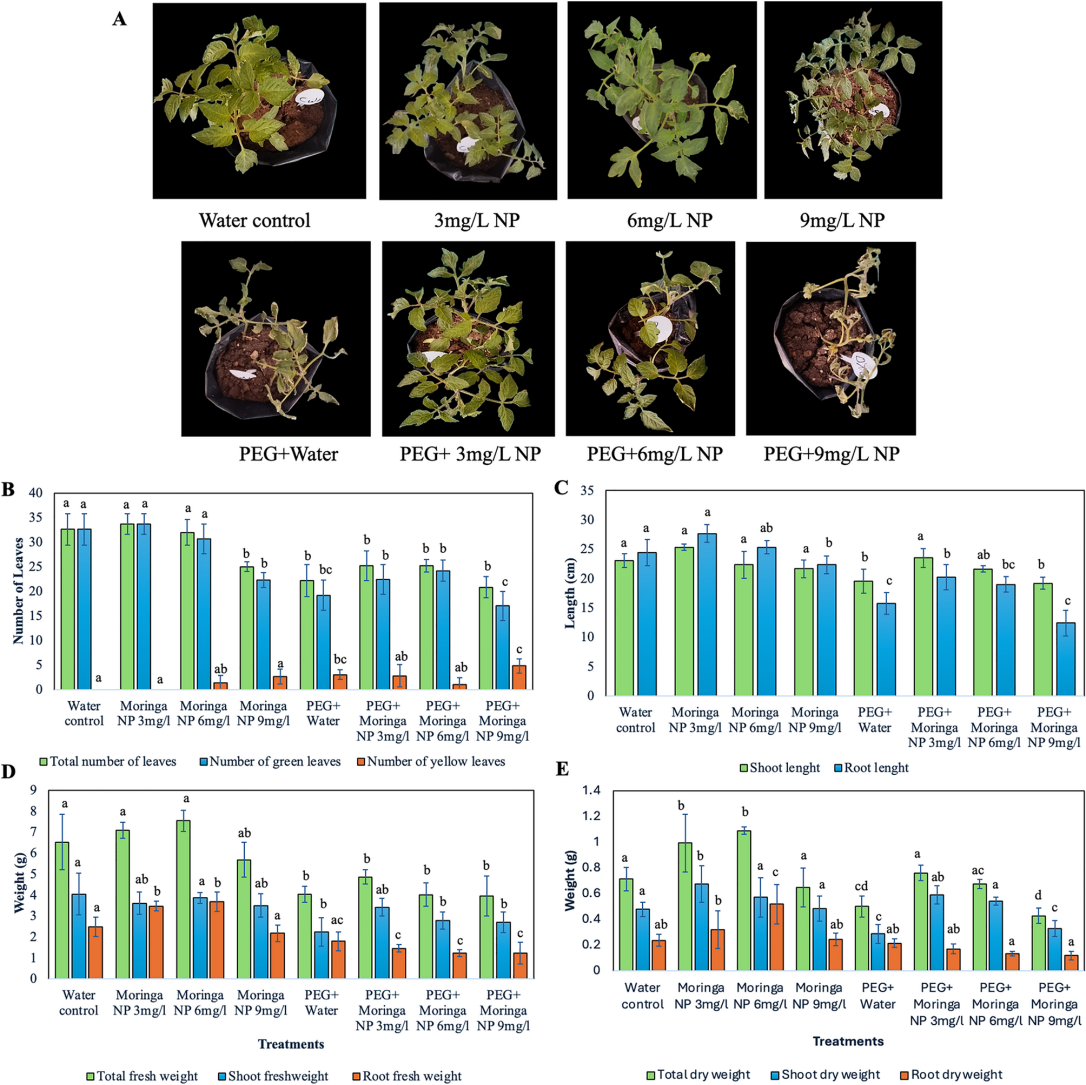
Growth performance and physiological parameters of tomato plants treated with CuO NPs under normal and drought conditions. (**A**) Morphological responses of plants under different treatments. Quantitative data on (**B**) leaf number, (**C**) shoot and root lengths, (**D**) fresh weight, and (**E**) dry weight. CuO NP treatments, particularly at 6 mg/L, improved growth and mitigated the drought effects induced by PEG. The error bars represent the standard error (SE). Different lower-case letters indicate significant differences (*p* < 0.05) between groups

This study evaluated the effects of the foliar application of copper oxide nanoparticles (CuO NPs) to tomato plants under drought stress and used polyethylene glycol (PEG) to simulate drought conditions. The results showed that while lower concentrations of CuO NPs (3 mg/L and 6 mg/L) promoted leaf health, higher concentrations (9 mg/L) led to stress, resulting in a 36% reduction in leaf count (20.8 ± 2.17) and a substantial increase in yellow leaves (4.8 ± 1.5) compared to the non-stress control (32.6 ± 3.2 and 0, respectively). PEG treatments helped mitigate stress effects, particularly when combined with lower NP concentrations, which closely matched those of the water control group (Fig. 6B). Compared to the water control under normal conditions (shoot: 23.02 ± 1.16 cm, root: 24.2 ± 2.25 cm), 3 mg/L CuO NPs increased shoot length to 25.33 ± 0.58 cm (10% increase) and root length to 27.66 ± 1.53 cm (14% increase), but 9 mg/L reduced growth. Under drought stress, 3 mg/L CuO NPs improved shoot length to 23.5 ± 1.58 cm (20% increase) and root length to 20.26 ± 2.12 cm (29% increase), while 6 mg/L CuO NPs improved shoot length to 21.62 ± 0.51 cm (11% increase) and root length to 19 ± 1.33 cm (21% increase) compared to the PEG control (shoot: 19.52 ± 2.05 cm, root: 15.72 ± 1.86 cm), emphasizing the need for optimized dosage (Fig. 6C).

Total fresh and dry biomass of tomato plants showed notable changes with CuO NP treatments. Under normal conditions, both 3 mg/L and 6 mg/L CuO NPs significantly increased biomass compared to the non-stress control (6.5 ± 1.3 g and 0.71 ± 0.09 g). Specifically, 3 mg/L increased fresh and dry biomass to 7.08 ± 0.38 g and 0.99 ± 0.22 g (9% and 39% increase, respectively), while 6 mg/L resulted in 7.54 ± 0.5 g and 1.09 ± 0.03 g (16% and 53% increase, respectively). In contrast, 9 mg/L (5.68 ± 0.8 g and 0.65 ± 0.15 g) led to a slight reduction in biomass (13% and 9% decrease, respectively). Under drought stress, lower concentrations of CuO NPs (3 mg/L and 6 mg/L) improved both fresh and dry weights compared to the drought stress control (4.03 ± 0.38 g and 0.5 ± 0.08 g). Specifically, at 3 mg/L, fresh and dry biomass was 4.9 ± 0.33 g and 0.76 ± 0.06 g (21% and 52% increase, respectively), and at 6 mg/L biomass was 4.02 ± 0.56 g and 0.67 ± 0.034 g (0% and 34% increase, respectively) (Fig. 6D and E). These findings highlight the potential of CuO NPs, particularly at moderate concentrations, to enhance tomato plant growth and resilience across different conditions.

**Fig. 7:**
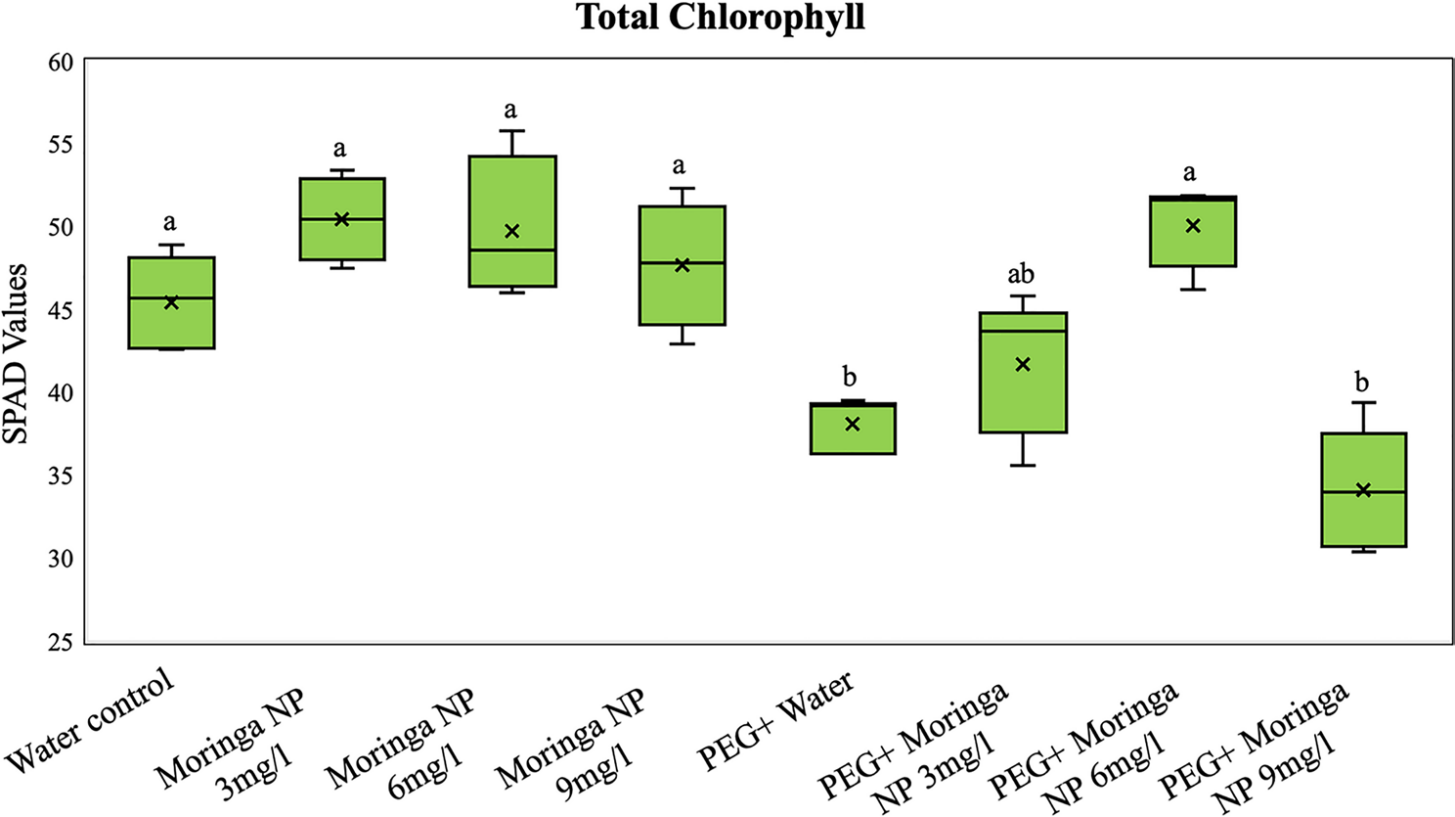
Effects of Moringa-based CuO NP and PEG treatments on the total chlorophyll content in plants under different conditions. Box plots showing SPAD values for the water control, moringa NP (3, 6, and 9 mg/L), PEG with water, and PEG combined with CuO NPs. Boxes represent the interquartile range, lines indicate medians, and ‘x’ marks the means. Whiskers depict variability, highlighting treatment effects on chlorophyll levels. Different lower-case letters indicate significant differences (*p* < 0.05) between groups

In this study, the effects of Moringa-based CuO NP and PEG treatments on the total chlorophyll content (measured as SPAD values) in plants were evaluated. The treatments included the following: water control; Moringa NP at concentrations of 3, 6, and 9 mg/L; PEG alone; and PEG combined with Moringa NP at the same concentrations (Fig. 7).

The results revealed that, under normal conditions, CuO NP application, particularly at a concentration of 3 mg/L, significantly increased the chlorophyll content to an average of 51.43 ± 1.6 (a 13% increase) compared with that of the water control (45.46 ± 2.8). However, the highest concentration (9 mg/L) resulted in no significant difference in chlorophyll levels, with mean 47.7 ± 4.7, indicating a possible threshold for optimal NP effectiveness. Compared with PEG alone (drought stress control 38.1 ± 1.65), PEG combined with CuO NPs also improved the chlorophyll content, with 6 mg/L being the most effective concentration, averaging 50.08 ± 2.47 (a 32% increase) (Fig. 7). These findings suggest that the photosynthetic pigment levels of Moringa plants can be improved and that their effectiveness is influenced by their concentration and combination of treatments.

### Relative gene expression of stress-responsive genes

We investigated the relative expression levels of Phenylalanine Ammonia-Lyase (*PAL*), Chalcone Synthase (*CHS*), and Hydroxycinnamoyl-CoA: Quinate Hydroxycinnamoyl Transferase (*HQT*) genes, which are critical in the plant stress response and secondary metabolism, under various treatments. Compared with the water control, theoringa-based CuO NPs significantly upregulated gene expression, with the highest expression observed at 9 mg/L for all genes. The combination of PEG and Moringa NPs further increased expression, surpassing the effect of either treatment alone, particularly at 9 mg/L. These findings suggest a synergistic effect of PEG and Moringa NPs in activating stress-related and phenylpropanoid pathway genes, potentially enhancing plant defense mechanisms.

**Fig. 8:**
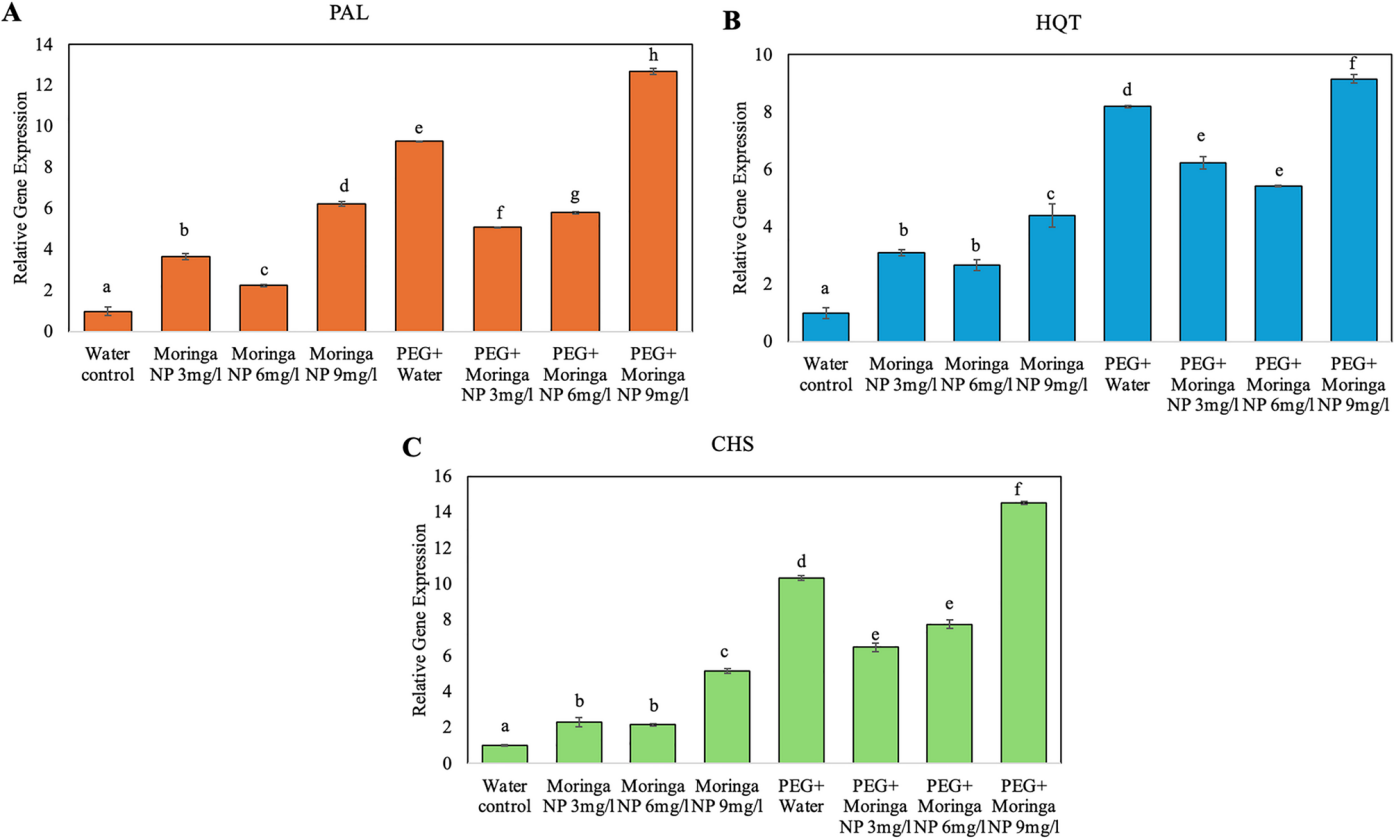
Relative expression of the *PAL*, *HQT*, and *CHS* genes under Moringa-based CuO NP and PEG treatments. (**A**) *PAL*, (**B**) *HQT*, and (**C**) *CHS* expression levels for the water control, Moringa NP (3, 6, and 9 mg/L), PEG, and PEG combined with CuO NPs. The bars represent the means ± standard errors, with different letters indicating significant differences (*p* < 0.05). The combined PEG and Moringa NP treatment group presented the highest gene expression, highlighting their role in the stress response

The expression of *PAL*, which is involved in the phenylpropanoid pathway and plant defense, was highest in the plants subjected to combinations of PEG (drought stress), Moringa extract, and nanoparticles (NP), suggesting an increased stress response. Similarly, *HQT*, which contributes to the synthesis of the antioxidant chlorogenic acid, exhibited peak expression under the PEG + Moringa + NP (6 mg/L) treatment, highlighting its role in activating antioxidant pathways during stress. *CHS*, which is essential for flavonoid biosynthesis and stress tolerance, also increased under treatment with Moringa and NPs, particularly under drought conditions (PEGs), indicating that these treatments may increase flavonoid production to mitigate stress. Overall, the data suggest that treatments involving Moringa extract and nanoparticles under drought stress synergistically activate key stress-related genes, suggesting a promising approach to improve tomato plant resilience, growth, and yield under adverse conditions (Fig. 8).

## Discussion

*Moringa oleifera*-delivered nanoparticles have recently gained the attention for their wide-ranging uses in medicine, agriculture, and environment. Known for their nutritional and medicinal benefits and the plant rich nature in multiple bioactive components. In agriculture, there has been limited research exploring the potential of Moringa-based nanoparticles to reduce reliance on chemical fertilizers. These nanoparticles, derived from *Moringa oleifera*, could offer a sustainable alternative for enhancing crop growth, soil health and abiotic stress tolerance. However, further studies are needed to fully understand their effectiveness and practical applications in farming systems. Their eco-friendly and cost-effective nature makes them a sustainable option for various applications [16, 33]. In this study, we examined the impact of foliar application of Moringa-based copper oxide nanoparticles on tomato plants under drought stress conditions. The research focused on evaluating how these nanoparticles influence plant growth, resilience, and overall performance during water scarcity. The findings aim to provide insights into sustainable strategies for improving crop tolerance to drought.

The synthesized copper oxide nanoparticles (CuO NPs) exhibited distinct optical, chemical, and structural properties, which is consistent with previous studies. For example, UV‒Vis spectrophotometry confirmed a surface plasmon resonance (SPR) peak at 285 and 340 nm, which aligns with the characteristic SPR range of CuO nanoparticles (200–350 nm) reported in earlier works [28, 31, 34]. FTIR analysis revealed functional groups such as O-H, C-H, C = C, S = O, and C-O stretching, indicative of a complex chemical composition similar to that of the green-synthesized CuO NPs described in prior research [35, 36]. Morphological characterization via SEM and TEM revealed nanoparticle sizes ranging from 26 to 33 nm and 26–55 nm, respectively. The broader size range observed via TEM likely reflects nanoparticle aggregation, a common phenomenon in nanoscale systems [37]. These size ranges are comparable to those reported in other plant-based CuO NP syntheses [38], confirming successful synthesis with appropriate physical attributes. The zeta potential of the synthesized Moringa CuO nanoparticles was found to be -19.5 mV, indicating a moderate stability in their dispersion. This stability is likely due to the repulsive electrostatic forces acting between particles, a phenomenon well-documented in colloidal systems [39, 40]. This finding is consistent with previous studies on nanoparticles derived from Moringa, which have shown comparable stability profiles [16]. This negative charge supports adsorption onto plant surfaces and may aid in penetration through cell walls and membranes. It also impacts cellular internalization and distribution within plant tissues [41]. At suitable concentrations, these nanoparticles can enhance plant growth, photosynthetic activity, antioxidant enzyme function, and nutrient absorption while offering antimicrobial benefits for plant protection [42]. However, their effectiveness is concentration-dependent, as excessive amounts may cause phytotoxicity. Thus, we confirmed the successful synthesis of CuO NPs with properties suitable for applications in agriculture, biomedicine, and environmental remediation, offering potential for further development in nanotechnology.

Furthermore, we demonstrated the potential of CuO nanoparticles (NPs) in enhancing tomato plant growth and stress tolerance. At moderate concentrations (3 mg/L and 6 mg/L), CuO NPs significantly improved leaf health, shoot and root length, biomass, and chlorophyll content under normal and drought conditions, which is consistent with previous findings in which nanoparticles increased photosynthesis and stress resistance in various crop species [43, 44]. The 6 mg/L concentration was most effective at mitigating drought-induced decreases in growth and chlorophyll levels, likely because of improved nutrient uptake and stress tolerance. However, higher concentrations (9 mg/L) resulted in reduced growth and chlorophyll content, likely due to oxidative stress and cellular disruption, as previously reported [45–47]. These findings emphasize the concentration-dependent effects of CuO NPs, with lower doses offering significant benefits and higher doses at risk of toxicity.

CuO NPs enhance drought tolerance in plants through multiple mechanisms. They improve water retention by interacting with plant cell walls and membranes, reducing water loss, regulating stomatal closure and potentially modifying the cell wall structure to hold more water [48, 49]. CuO NPs also alleviate oxidative stress by increasing the activity of antioxidant enzymes, such as superoxide dismutase (SOD) and catalase (CAT), which protect plants from reactive oxygen species [50]. Furthermore, CuO NPs increase photosynthetic efficiency, even under drought conditions, ensuring continued energy production [51]. These nanoparticles also upregulate genes involved in osmotic regulation and stress response, improving drought resilience [52]. CuO NPs promote root growth, allowing deeper water absorption and facilitating nutrient uptake, contributing to overall plant health and drought tolerance [53, 54]. Although these mechanisms are well known, CuO NPs may also exhibit phytotoxicity [55], making it essential to conduct experiments to optimize their dosage.

To gain a deeper understanding of the molecular mechanism by which CuO NPs influence plant growth under normal conditions—specifically regarding stress responses and metabolic pathways—three key genes were selected for study: *PAL*, *CHS*, and *HQT*. These genes are crucial for the response of tomato plants to drought stress. *EF1-α* was utilized as an internal standard in our gene expression analysis because of its stable expression across various tissues and under various environmental conditions, including drought. In the context of tomato plants, *EF1-*α provides a reliable reference for normalizing qRT‒PCR data, ensuring precise and consistent measurement of target gene expression [56].

Our investigation into the relative expression of *PAL*, *HQT*, and *CHS* corroborates and expands upon previous findings related to plant stress physiology and secondary metabolite biosynthesis. The activation of these genes in the phenylpropanoid pathway underscores their importance in helping plants withstand various abiotic stresses, thus contributing to their overall resilience.

This study demonstrates a significant upregulation of *PAL*, *CHS*, and *HQT* genes under combined stress from drought (induced by PEG treatment) and CuO NPs (6 mg/L). Numerous studies have highlighted the regulation of *PAL*, *HQT*, and *CHS* under different stress conditions. These genes are essential components of the phenylpropanoid pathway, crucial for plant defenses against diverse biotic and abiotic stresses. PAL initiates this pathway, leading to the synthesis of important phenolic compounds, while CHS and HQT produce antioxidants and stress-related metabolites [57–61]. These findings collectively emphasize the critical role of the phenylpropanoid pathway in enhancing plant resilience under stress such as drought. The upregulation of those genes aligns with growing evidence that CuO NPs enhance plant physiological responses to environmental stress. Research indicates that these metal-based NPs can improve drought tolerance by modulating gene expression [62]. Notably, CuO NPs have been linked to enhanced antioxidant systems and modulation of stress response genes across various species, including barley [63]. The observed synergistic effect, where combined application of CuO NPs and PEG amplifies gene expression, suggests these nanoparticles prime the plant’s natural defense mechanisms. This provides a strong molecular basis for improved physiological parameters and drought tolerance in tomato plants treated with Moringa-synthesized CuO NPs. These findings collectively emphasize the critical role of the phenylpropanoid pathway in enhancing plant resilience under stress.

Our results indicate that, compared with water, Moringa nanoparticles (NPs) notably increased the expression levels of these genes. This finding aligns with existing research that has demonstrated how nanoparticles can increase plant stress tolerance through gene expression modulation [43]. The peak expression observed at 9 mg/L across all genes indicates an optimal concentration of Moringa NPs for promoting gene activation, which resonates with other studies that document dose-dependent plant responses to nanoparticles [64].

Furthermore, the combination of PEG with Moringa-based CuO NPs further amplified gene expression. These findings support earlier findings on the synergistic effects of combined treatments. Since PEG is often used to simulate drought stress, its influence on gene expression is well established [65]. The enhanced expression resulting from the joint application of PEG and Moringa-based CuO NPs—greater than either treatment alone—suggests that Moringa-based CuO NPs may strengthen the ability of plants to cope with osmotic stress [66]. This synergistic effect echoes other studies that show how plant growth-promoting nanoparticles can work in tandem with abiotic stressors to activate stress-related pathways more effectively than when applied individually [44].

Cytotoxicity analysis is crucial for ensuring the safety and effectiveness of the nanoparticles in use. Since our study focuses on applying Moringa-synthesized copper oxide nanoparticles to tomato plants under drought stress, it is vital for future research to assess whether these nanoparticles harm against plant cells at the tested concentrations. Previous studies have investigated the cytotoxicity of silver synthesized moringa nanoparticles on mammalian cell lines [67–69]. However, the cytotoxic effect of Moringa-synthesized against plant cells have not been explored. Only by evaluating the cellular responses, we can provide valuable a wider insight into the safe and efficient use of nanotechnology in improving crop resilience under challenging environmental conditions.

## Conclusion

This study investigated the impact of Moringa-synthesized copper oxide nanoparticles (CuO NPs) on tomato plants subjected to drought stress. Results indicated that CuO NPs, particularly at concentrations of 3 and 6 mg/L, improved plant performance under drought conditions, as evidenced by enhanced growth and reduced leaf yellowing. Analysis of gene expression revealed changes in key stress-response genes (*PAL*, *CHS*, *HQT*), suggesting a role for these genes in mediating the observed drought tolerance. The combination of drought stress and CuO NP treatment appeared to further influence these genes. These findings suggest Moringa-synthesized CuO NPs could be a useful tool for improving tomato production in water-limited environments. Future research should focus on elucidating the precise molecular mechanisms by which CuO NPs influence the expression of these stress-response genes. Additionally, field trials are needed to validate these findings under real-world agricultural conditions and to assess the long-term effects of CuO NP application on plant health and soil microbial communities.

## Electronic supplementary material

Below is the link to the electronic supplementary material.

Supplementary Material 1

## Data Availability

The data that support the findings of this study are available within the manuscript or supplementary information.

## Abbreviations

ANOVA: Analysis of variance
PEG: Polyethylene glycol
NPs: Nanoparticles
CuO: Copper Oxide
EF1-α: Elongation Factor 1-alpha
PAL: Phenylalanine Ammonia-Lyase
HQT: Hydroxycinnamoyl-CoA Quinate Hydroxycinnamoyl Transferase
CHS: Chalcone Synthase
qRT-PCR: Quantitative real time PCR
SD: Standard deviation
SEM: Scanning electron microscopy
TEM: Transmission electron microscopy
FTIR: Fourier transform infrared
UV-vis: Ultra-violet visible
SPAD: Soil Plant Analysis Development
