# Supplementary material for: Moringa (Moringa oleifera) green-synthesized copper oxide nanoparticles for the drought tolerance of tomato (Solanum lycopersicum)
Source: BMC Plant Biol. 2025 May 23;25:685. doi: 10.1186/s12870-025-06708-2 (PMC12100965; doi:10.1186/s12870-025-06708-2)
Supplement: Supplementary file 1 — Supplementary Material 1 [file 12870_2025_6708_MOESM1_ESM.docx]

**
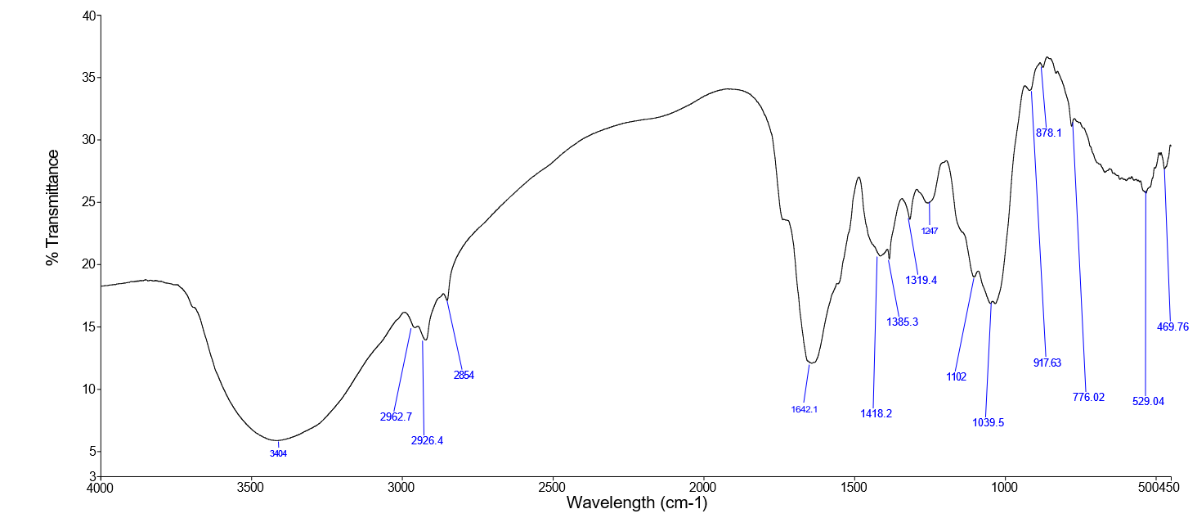
**

**Supplementary Fig. 1:** Fourier transforms infrared (FT-IR) spectra analysis of Moringa dried leaves showing the presence of various functional groups that can act as reducing and stabilizing agents for the formation of copper oxide nanoparticles.

**Supplementary Table 1:** Absorption peaks of the Moringa dried leaves obtained from the FTIR spectrophotometer and their corresponding groups.

| No. | Absorption Peak Position (Wavenumber cm-1) | Functional Group* |
| --- | --- | --- |
| 1 | 3404 | O-H stretching |
| 2 | 2962.7 - 2926.4 - 2854 | C-H stretching |
| 3 | 1642.1 | C=C stretching |
| 4 | 1418.2 - 1385.3 - 1319.4 | C-H bending |
| 5 | 1247 – 1102 - 1039.5 | C-O stretching |
| 6 | 917.63 - 878.1 - 776.02 | C-O stretching |
| 7 | 529.04 - 469.76 | C-Br, C-I stretching |

*FTIR Functional Group Database Table with Search InstaNANO.
